# Can vitamin D be an adjuvant therapy for juvenile rheumatic diseases?

**DOI:** 10.1007/s00296-023-05411-5

**Published:** 2023-08-11

**Authors:** Maciej K. Stawicki, Paweł Abramowicz, Gabriela Sokolowska, Sebastian Wołejszo, William B. Grant, Jerzy Konstantynowicz

**Affiliations:** 1 Department of Pediatrics, Rheumatology, Immunology, and Metabolic Bone Diseases, Medical University of Bialystok, University Children’s Clinical Hospital in Bialystok, Waszyngtona Street 17, 15274 Bialystok, Poland; 2 University Children’s Clinical Hospital in Bialystok, Bialystok, Poland; 3 Sunlight, Nutrition, and Health Research Center, San Francisco, CA USA

**Keywords:** Vitamin D, Juvenile idiopathic arthritis, Juvenile systemic lupus erythematosus, Juvenile systemic scleroderma, Idiopathic inflammatory myopathies, Periodic fever syndromes

## Abstract

Vitamin D, known for its essential role in calcium and bone homeostasis, has multiple effects beyond the skeleton, including regulation of immunity and modulation of autoimmune processes. Several reports have shown suboptimal serum 25 hydroxyvitamin D [25(OH)D] levels in people with different inflammatory and autoimmune rheumatic conditions, and an association between 25(OH)D levels, disease activity and outcomes. Although most available data pertain to adults, insights often are extended to children. Juvenile rheumatic diseases (JRDs) are a significant health problem during growth because of their complex pathogenesis, chronic nature, multisystemic involvement, and long-term consequences. So far, there is no definitive or clear evidence to confirm the preventive or therapeutic effect of vitamin D supplementation in JRDs, because results from randomized controlled trials (RCTs) have produced inconsistent outcomes. This review aims to explore and discuss the potential role of vitamin D in treating selected JRDs. Medline/PubMed, EMBASE, and Scopus were comprehensively searched in June 2023 for any study on vitamin D supplementary role in treating the most common JRDs. We used the following keywords: “vitamin D” combined with the terms “juvenile idiopathic arthritis”, “juvenile systemic scleroderma”, “juvenile systemic lupus erythematosus”, “juvenile inflammatory myopathies”, “Behcet disease”, “periodic fever syndromes” and “juvenile rheumatic diseases”. Observational studies have found that serum 25(OH)D concentrations are lower in juvenile idiopathic arthritis, juvenile systemic lupus erythematosus, juvenile systemic scleroderma, Behcet disease and proinflammatory cytokine concentrations are higher. This suggests that vitamin D supplementation might be beneficial, however, current data are insufficient to confirm definitively the complementary role of vitamin D in the treatment of JRDs. Considering the high prevalence of vitamin D deficiency worldwide, children and adolescents should be encouraged to supplement vitamin D according to current recommendations. More interventional studies, especially well-designed RCTs, assessing the dose–response effect and adjuvant effect in specific diseases, are needed to determine the potential significance of vitamin D in JRDs treatment.

**Supplementary Information:**

The online version contains supplementary material available at 10.1007/s00296-023-05411-5.

## Introduction

Vitamin D’s role in the human body extends beyond maintaining the skeleton’s mineral balance. A secosteroid hormone, vitamin D exerts its functions via the vitamin D receptor (VDR), a transcription factor found in the skin, muscle, skeleton, kidney, adipose tissue, pancreas, blood vessels, brain, breast tissue, placenta, and immune cells. Vitamin D not only is synthesized mainly in the skin through sun exposure but also is obtained through supplements and, to a lesser extent, dietary sources such as fish and fortified foods [1].

Vitamin D has been extensively studied in various health issues, including chronic and infectious diseases. Although the evidence regarding its significance and preventive role is not entirely consistent, well-designed studies generally support the notion that vitamin D offers several health benefits across the stages of life [2–4].

Juvenile rheumatic diseases (JRDs) encompass a range of conditions affecting joints, tendons, muscles, ligaments, and bones, as well as vital organs such as the lungs, heart, and kidneys. The exact causes of most JRDs and connective tissue diseases are not fully understood, and their etiology is believed to be multifactorial [5]. Therefore, treatment of those conditions often targets multiple mechanisms or symptoms. Management protocols for JRDs are disease-specific, and many therapies rely on classical or biological disease-modifying antirheumatic drugs (DMARDs). Given the chronic nature of those conditions, which affect children’s present and future health, managing JRDs typically involves long-term treatment strategies that require regular monitoring of therapeutic outcomes, advantages, and potential adverse reactions [6].

Previous researchers have investigated how adequate serum 25-hydroxyvitamin D [25(OH)D] concentration affects juvenile and adult rheumatic diseases. However, findings have been inconsistent and data regarding the pediatric population remain limited [7–10]. Likewise, beneficial effects of vitamin D intake and supplementation have been observed in patients with various chronic diseases, including those with rheumatic diseases [11–14]. Vitamin D’s positive influence on disease progression and outcomes is probably due to its role in maintaining bone turnover, calcium homeostasis, muscle function, and mineral metabolism, as well as regulating immune and inflammatory responses [4, 15]. The existing literature emphasizes the importance of maintaining high or optimal concentrations of serum 25(OH)D in shaping the clinical manifestations of rheumatic diseases, although the available data focus mainly on the adult population. Vitamin D’s potential therapeutic role in JRD remains poorly understood.

This review aims to assess vitamin D’s specific role as a treatment component or therapeutic agent in JRDs. We sought to gather and summarize the available evidence on vitamin D supplementation in selected juvenile rheumatic conditions.

## Methods

Medline/PubMed, EMBASE, and Scopus were comprehensively searched in June 2023 for any study on vitamin D supplementary role in treating the most common JRDs. We used the following keywords: “vitamin D” combined with the terms “juvenile idiopathic arthritis”, “juvenile systemic scleroderma”, “juvenile systemic lupus erythematosus”, “juvenile inflammatory myopathies”, “juvenile rheumatic diseases”, “Behcet disease”, “periodic fever aphthous stomatitis pharyngitis and adenopathy syndrome”, “familial Mediterranean fever”, “hyper-IgD syndrome”, “cryopyrin-associated periodic syndrome”, “tumor necrosis factor receptor-associated periodic syndrome”, “periodic fever syndromes”. The search strategies for each database can be found in Appendix. No publication date restriction was applied. Only papers in English regarding populations younger than 18 years were taken into consideration. Most recent and most cited publications were in favor. In addition, we screened the reference lists of the selected publications to ensure that no potentially relevant studies were missed. Forward references searching of included studies was conducted using Web of Science to identify other research that has referenced any article of interest. Finally, all identified articles were screened for eligibility, first based on their title and abstract, followed by a review of their full text carried out by two independent reviewers (MS and GS), and any conflicts were resolved by consensus.

## Vitamin D: chemistry, metabolism, optimal serum concentration, and supplementation guidelines

Cholecalciferol, vitamin D, is produced in the epidermis from 7-dehydrocholesterol upon exposure to solar energy. Solar UVB radiation (wavelength, 290–315 nm) breaks the chemical bond between carbon atoms 9 and 10, forming previtamin D_3_, which is later converted into chemically stable vitamin D_3_, known as cholecalciferol. After attaching to the transporting molecule, vitamin D-binding protein (VDBP), cholecalciferol travels to the liver. Excessive sun exposure does not result in overproduction or intoxication of vitamin D; sunlight decomposes any surplus. Organic synthesis is the richest source of vitamin D, but it also can be obtained through dietary intake (e.g., fish, meat, offal, mushrooms, eggs, fortified food) and supplementation [1, 16].

When reaching the liver, cholecalciferol is converted by oxidases related to cytochrome P450 (CYP2R1 is believed to be the first 25-hydroxylase) into calcidiol [25(OH)D]. From the human liver, the VDBP–25(OH)D complex is then transported to the kidneys, where 1α-hydroxylase Cyp27B1, another CYP450-related enzyme, maintains conversion to calcitriol [1,25(OH)_2_D], the biologically active form of vitamin D. Calcitriol increases the intestinal absorption of calcium and phosphate, increases expression of fibroblast growth factor 23, and decreases parathyroid hormone (PTH) production. All the above factors control calcitriol production in feedback mechanisms [15, 16]. Increased expression of 25-hydroxyvitamin D-24-hydroxylase, which catabolizes calcitriol to an inactive form, self-regulates the concentration of calcitriol.

Vitamin D’s positive influence on skeletal health is well-known and thoroughly described. Vitamin D plays a major protective role against rickets and osteomalacia [4]. It helps regulate calcium homeostasis and has a positive influence on bone mineral density (BMD) [7]. Given that VDR and CYP27B1 are present in many cell types [17], vitamin D has a vast range of potential nonskeletal effects. It is related to the proliferation and differentiation of epidermal cells and improves wound healing [18]. Evidence also exists to substantiate vitamin D’s beneficial effects in reducing cardiovascular risk factors and the frequency of cardiovascular events [14]. Unfortunately, those findings do not fully correspond to the results of interventional studies owing to their incorrect design [19, 20].

Skin synthesis is a major source of vitamin D [21]. However, its production can be limited by factors such as weather conditions and geographical location. For example, in Central Europe, sufficient sun availability for vitamin D synthesis occurs only between late April and early September [22]. Other limitations include skin type, age, use of sunscreens that can reduce UVB radiation absorption by 90–95%, cancer awareness, and wearing clothing that covers arms and legs [21].

The concentration of 25(OH)D is considered the most appropriate indicator of vitamin D status, representing both synthesized and supplemented vitamin D with a half-life of 2–3 weeks. Current recommendations define concentrations below 20 ng/ml as deficient and concentrations of 21–29 ng/ml as insufficient [23, 24]. An optimal concentration is considered to be between 30 and 50 ng/ml, with levels above 30 ng/ml being significant and sufficient for positive effects on the skeletal system [16, 24]. Discussions are ongoing regarding whether that concentration of 25(OH)D also confers full extraskeletal effects [4, 25]. Despite vitamin D’s widespread and positive influence on human health, population studies reveal that vitamin D deficiency remains a global concern [16, 26–28].

Supplementation and treatment of vitamin D deficiency are regularly reevaluated. According to current recommendations, daily supplementary and therapeutic doses range from 400 to 2000 IU/day [23–29], which can achieve a concentration of 25(OH)D above 29 ng/ml. Several studies have confirmed the safety and lack of observed side effects for higher doses [30, 31]. Recommendations emphasize that dosage should be adjusted based on individual patient factors, including comorbidities and obesity. Doses should be modified accordingly, considering both the increased requirement for vitamin D and the potential toxic effects of excessive doses. Pending reliable data from well-designed clinical trials, general recommendations for the healthy population are typically followed.

### Multipotential and pleiotropic effects of vitamin D

Vitamin D’s biological actions are moderated by the VDR, a transcription factor expressed not only in muscle and bone cells but also in a spectrum of human tissues including immune cells [32]. Vitamin D-activated VDR is believed to have multiple binding sites throughout the genome with the possibility to affect the regulation of multiple gene transcription [15, 32, 33]. The genetic process by which 1,25(OH)_2_D exerts its effects includes the direct attachment of activated VDR to particular DNA sequences known as vitamin D response elements located near target genes. That binding can either activate or suppress transcription [33]. In addition, vitamin D exerts rapid effects that occur independently of gene transcription. Those nongenomic actions involve binding to membrane receptors, regulating calcium homeostasis, influencing ion channels, influencing cellular differentiation and proliferation, exhibiting anti-inflammatory effects, and potentially affecting neurological processes [34].

#### Vitamin D and bones

Among vitamin D’s various functions in the body, the most essential is to maintain proper skeletal balance. Vitamin D is responsible mainly for maintaining adequate calcium levels. When serum calcium decreases, PTH secretion is stimulated, leading to the synthesis of calcitriol. Calcitriol enhances intestinal calcium absorption, which depends on dietary intake, solubility, and intestinal capacity [35]. Both PTH and calcitriol promote calcium reabsorption in the kidneys’ distal tubules while inhibiting phosphate reabsorption, directly or indirectly [36].

Vitamin D and PTH also induce calcium mobilization. Along with interleukin 6 (IL-6), they stimulate osteoblasts to express the receptor activator of NF-κB ligand (RANKL). RANKL interacts with the RANK receptor on osteoclast progenitor cells, facilitating osteoclast maturation and later activation. Osteoblasts produce osteoprotegerin, a decoy receptor for RANKL, which helps regulate the osteoclastic response [37]. Activated osteoclasts are responsible for bone resorption, leading to increased serum calcium levels [38]. The elevated calcium level, resulting from increased intestinal absorption and bone resorption, activates receptors that reduce PTH production and then decrease 1,25(OH)_2_D. In addition to increasing bone resorption, vitamin D also restricts bone mineralization, enhancing calcitriol’s hypercalcemic effect [34].

Furthermore, prolonged vitamin D deficiency, leading to deficient phosphate levels, is proven to disrupt the balance of cartilaginous growth plates in animal models [39, 40]. Dysregulation of hypertrophic chondrocyte apoptosis promotes cartilage expansion and widening by reducing chondrocyte apoptosis, resulting in delayed growth plate mineralization in children. Moreover, decreased vascular endothelial growth factor and RANKL restrict vascularization as well as the production and differentiation of chondrocytes and osteoclasts [41].

Conversely, vitamin D exerts opposite effects when calcium levels are sufficient. Stimulating the VDR promotes mature osteoblasts, leading to anabolic and antiresorptive effects and increased bone mass. The antiresorptive effect is mediated by a decreased RANKL/osteoprotegerin ratio, whereas the anabolic effect may be associated with increased expression of LRP5 [34].

The physiological significance of the diverse and sometimes opposing effects of VDR signaling in osteogenic cells is still not fully understood and requires further investigation.

#### Vitamin D and immunity

Vitamin D exerts a significant influence on the immune system and plays a crucial role in modulating both innate and adaptive immunity in various ways. The active form of vitamin D, 1,25(OH)_2_D, hinders the adaptive immune response while enhancing innate immunity. Additionally, local production of 1,25(OH)_2_D by monocytes and macrophages, leads to the production of immunoglobulins by B lymphocytes and reduces the synthesis of autoimmune antibodies [34]. Vitamin D also plays a beneficial role in stabilizing endothelial membranes [42].

Many studies have shown vitamin D’s preventive effects against bacterial and viral infections, achieved by inducing the production of the antimicrobial peptide cathelicidin (LL-37) and reactive oxygen species [3, 43]. That mechanism is particularly crucial, because infections pose a significant risk for autoimmune diseases [44].

In immune cells, intracrine production of 1,25(OH)_2_D influences adaptive immunity by inhibiting T-cell-driven inflammation and transforming dendritic cells into a more tolerogenic state, characterized by the production of IL-10. Consequently, dendritic cells’ ability to present antigens and activate T cells decreases [45]. In addition, 1,25(OH)_2_D suppresses the production of IL-12, IL-23, and IL-6, thereby inhibiting the development of Th1 cells that produce gamma interferon (IFN-γ) and IL-2, as well as Th17 cells that produce IL-17 [46].

Furthermore, an increase occurs in the production of T regulatory cells that exert suppressive effects on inflammatory processes [47–49]. Those diverse effects of vitamin D make it an intriguing subject for researchers investigating autoimmune diseases.

#### Other extraskeletal effects of vitamin D

Because VDR is expressed in a variety of human cells, different effects of vitamin D are observed. Many are under investigation without firm conclusions. Although available data remain scarce, administering standard doses of vitamin D to vitamin D-deficient older patients seems to improve muscle function and play a beneficial role in decreasing the incidence of falls [50, 51]. In addition, vitamin D’s contribution to bone health, which supports muscles, can help prevent fractures. More well-designed trials are required to establish the optimal serum 25(OH)D concentration and dosage for vitamin D to exert a positive muscular effect.

Both in vitro and in vivo studies postulate the relationship between vitamin D and cancer, especially colon cancer. Low vitamin D status is linked with several cancers, whereas vitamin D influences cell maturation, differentiation, and apoptosis [34, 52]. As well as being involved in angiogenesis, vitamin D can regulate the metastatic potential of many tumors [53, 54]. Unfortunately, even large clinical studies failed to prove that vitamin D supplementation is related to a lower incidence of cancer or better outcomes. Some of those failures were due to inappropriate study design [52, 54, 55]. Also, Mendelian randomization studies reported no relationship between serum 25(OH)D and cancer incidence except for ovarian cancer [56].

A strong connection also is evident between a complete absence of vitamin D action and negative cardiovascular effects arising from preclinical studies, including biochemical, genetic, and animal data. Vitamin D can decrease the risk of cardiovascular disease by mitigating factors such as vascular inflammation, endothelial dysfunction, smooth muscle cell proliferation, hypertension, and secondary hyperparathyroidism [57]. Although some intervention studies conducted confirmed a positive association between higher 25(OH)D concentration and better control of systolic and diastolic blood pressure [58], others produced conflicting results [59]. The causal nature of the associations between vitamin D and cardiovascular disease remains uncertain, and further research is needed to explore potential differences across patient populations [19].

### Effects of vitamin D on juvenile rheumatic diseases

Beginning in childhood or adolescence, JRDs encompass a spectrum of diseases that affect the connective tissue and musculoskeletal system. All such diseases share similar symptomatology, although each has specific symptoms [5]. Although the pathomechanisms of those diseases have not been fully understood, genetic, environmental, and immune-related mechanisms are believed to contribute to the pathogenesis. JRDs are originally associated mainly with arthritis, but the possibility of systemic involvement varies depending on the type of disease [6, 60].

As mentioned, vitamin D deficiency is a growing problem in the world’s population, including children and adolescents though biogeographical, ecological and ethnic factors may also matter leading to some differences. Global guidelines indicate the need for vitamin D supplementation and suggest optimal supplementation doses for specific age and risk groups, with cholecalciferol being the first choice. Most guidelines also suggest an upper dose level and recommend that supplementation be monitored through serum 25(OH)D concentrations. Also, because of the lack of detailed data, many guidelines suggest that in some cases, such as prolonged therapy with steroids, doses be consistently increased two to three times. Those recommendations also apply to the pediatric population [23, 24].

This section will focus only on the most frequent representative diseases included in the whole group of JRDs. We aimed to present representative, but not exhaustive, data about possible associations between vitamin D and JRDs (Table 1).

**Table 1:** Published data regarding the status of vitamin D and its therapeutic potential in selected rheumatic diseases in pediatric population

Ref.	Study design	Results
Juvenile idiopathic arthritis		
Tang et al. (2019) [71]	RCT	Supplementation with 2000 IU of cholecalciferol daily for 24 weeks significantly increased serum 25(OH)D levels in the study group compared with the control group. No differences according to BMD, and disease activity assessed with JADAS-27 were found between groups
Chiaroni-Clarke et al. (2019) [68]	Retrospective	Higher prediagnosis UVR exposure was associated with a lower risk of JIA, with a dose–response relationship. UVR exposure at 12 weeks of pregnancy was inversely linked to JIA. Lower UVR exposure may increase JIA risk
Thorsen et al. (2017) [69]	Case–control	No significant association was found between 25(OH)D levels and the risk of JIA. No evidence that 25(OH)D levels around birth are linked to later JIA risk
Clarke et al. (2023) [70]	Mendelian randomization	No evidence indicating a causal relationship between genetically predicted 25(OH)D levels and the incidence of JIA. Lack of evidence suggesting that genetically predicted JIA causally affects 25(OH)D levels
Nandi et al. (2022) [72]	Observational	A significant negative correlation between the JADAS-27 score and serum vitamin D was confirmed. The corrected Chi-square test showed a significant association between serum vitamin D status and disease activity groups
Sengler et al. (2018) [73]	Observational	~ 50% of the patients had inadequate 25(OH)D levels (< 20 ng/ml) in the initial serum sample, whereas 25% had inadequate levels in both samples. Inverse correlation between serum 25(OH)D, disease activity, and risk of developing JIA-associated uveitis
Çomak et al. (2014) [74]	Retrospective	Significant negative correlation between vitamin D levels and disease activity. Patients with 25(OH)D levels < 15 ng/ml had significantly higher mean JADAS-27 scores than patients with 25(OH)D levels > 15 ng/ml
Dağdeviren-Çakır et al. (2016) [76]	Cross-sectional	No significant difference in vitamin D levels between activation and remission periods in JIA patients. No significant association between disease activity and serum 25(OH)D. Significantly lower vitamin D levels in JIA and FMF children compared with healthy controls. Patients with JIA and FMF often showed vitamin D deficiency and insufficiency
Bouaddi et al. (2014) [77]	Cross-sectional	75% of patients exhibited hypovitaminosis D. Univariate analyses confirmed a negative correlation between 25(OH)D and DAS-28 scores in polyarticular and oligoarticular JIA. No significant association was found between 25(OH)D levels and BASDAI scores in juvenile spondyloarthropathy. Multivariate linear regression did not confirm any association between 25(OH)D levels and DAS-28 scores
Juvenile systemic lupus erythematosus		
Lima et al. (2016) [88]	RCT	Patients with JIA supplemented with a weekly dose of 50,000 IU of cholecalciferol for 24 weeks presented not only higher serum 25(OH)D levels but also a significant improvement in SLEDAI, ECLAM, and fatigue reduction compared with controls
Lima et al. (2018) [89]	RCT	Significantly higher trabecular number with a decrease in trabecular separation at the tibia site was observed in patients supplemented with 50,000 IU of cholecalciferol per week compared with controls
Abo-Shanab et al. (2021) [83]	Cross-sectional	jSLE patients showed significantly higher levels of IFN-γ and IL-17, and significantly lower levels of 25(OH)D than in controls. Negative correlation between 25(OH)D and both SLEDAI-2K and IFN-γ
Stagi et al. (2014) [84]	Cross-sectional	jSLE patients, especially with active disease, had lower 25(OH)D levels than controls. Moreover, reduced total calcium levels, increased phosphate levels, and higher BSAP and PTH were observed. jSLE patients had lower spine BMAD SDS values than controls, with higher values in patients with 25(OH)D sufficiency and insufficiency than in those with deficiency (*p* < 0.001)
Tabra et al. (2020) [85]	Cross-sectional	Significant differences in mean 25(OH)D concentrations between patients and controls, with significant differences between active and inactive patients. Significant negative correlations between serum 25(OH)D and SLEDAI, steroid dose, anti-dsDNA, 24-h proteinuria, and PTH. Significant positive correlations between 25(OH)D and C3, C4, serum calcium, and *Z* score, whereas nonsignificant correlations were found between 25(OH)D and serum phosphorus, disease duration, and steroid duration
Caetano et al. (2015) [86]	Observational	No significant difference in FMI, LMI, or ZBMI between measurements from the two-time points was found. Significant decrease in BMD in patients without vitamin D supplementation. Nearly half of the patients had altered nutritional status
Peracchi et al. (2014) [87]	Cross-sectional	Patients with JSLE exhibited significantly lower mean levels of calcium, albumin, and alkaline phosphatase compared to controls. Inadequate serum 25(OH)D concentrations were more often observed in jSLE patients than in controls. No associations were found with disease activity, PTH levels, alkaline phosphatase levels, medication use, or alterations in BMD
Behçet disease		
Can et al. (2012) [122]	Observational	BD patients commonly exhibited reduced levels of serum 25(OH)D. Vitamin D replacement had beneficial effects on endothelial function and led to a significant improvement in CIMT. Although FMD measurements also improved, the improvement did not reach statistical significance
Omar et al. (2022) [124]	Case–control	The study found that lower vitamin D levels in BD patients are associated with increased oxidative stress. Vitamin D levels were inversely correlated with disease activity, inflammation markers, and oxidative stress markers, while positively correlated with antioxidant levels
Güngör et al. (2016) [125]	Case–control	Vitamin D-deficient patients had significantly higher baseline plasma levels of ESMs compared to vitamin D sufficient patients, but there were no significant differences in baseline TLRs levels between the two groups. After vit D replacement, the mean plasma levels of ESMs significantly decreased, while the mean plasma levels of TLRs showed a decrease, but it was not significant. The active stage disease rate was slightly higher in the pre-treatment group compared to the post-treatment group, but the difference was not significant
Hamzaoui et al. (2010) [126]	Cross-sectional	Patients with active BD had lower vitamin D levels compared to both inactive BD patients and healthy controls. In active BD, vitamin D levels were found to be correlated with CRP and ESR levels. Additionally, serum vitamin D levels showed a positive correlation with the number of Treg cells. On the other hand, the Th1/Th2 ratio was inversely correlated with serum 25(OH)D levels
Zhong et al. (2021) [128]	Mendelian randomization	The analysis using inverse variance weighted estimate revealed that a genetically increased 25(OH)D level was linked to a higher risk of Behçet’s disease
Periodic fever, aphthous stomatitis, pharyngitis and adenitis syndrome (PFAPA)		
Stagi et al. (2014) [135]	Interventional study	PFAPA patients displayed lower 25(OH)D levels compared to controls. Winter 25(OH)D levels were notably reduced compared to those measured in summer and were significantly lower than in healthy controls. Serum 25(OH)D levels showed correlations with both fever episodes and C-reactive protein values. Following repletion, PFAPA patients experienced a significant decrease in the number and duration of febrile episodes
Familial Mediterranean fever (FMF)		
Kazem et al. (2021) [138]	cross-sectional	Following a 6-month dietary intervention involving Curcumin, vitamin D, and flaxseed, FMF patients in the study group experienced significant improvements in clinical presentation, cognitive test results, and overall well-being. The intervention also led to a notable increase in serum 25(OH)D levels and a decrease in CRP levels

*25(OH)D* 25-hydroxyvitamin D, *anti-dsDNA* anti-double-stranded DNA, *BASDAI* Bath ankylosing spondylitis disease activity index, *BMAD SDS* bone mineral apparent density standard deviation score, *BMD* bone mineral density, *BSAP* bone-specific alkaline phosphatase, *CIMT* Carotid Intima-Media Thickness, *CRP* C-reactive protein, *ECLAM* European consensus lupus activity measurement, *ESM* Endothelial selectine molecules, *ESR* erythrocyte sedimentation rate, *FMD* brachial artery flow mediated dilatation, *FMF* Familial Mediterranean Fever, *FMI* fat mass index, *IFN-γ* gamma interferon, *IL-17* interleukin 17, *JIA* juvenile idiopathic arthritis, *jSLE* juvenile systemic lupus erythematosus, *LMI* lean mass index, *PTH* parathyroid hormone, *RCT* randomized controlled trial, *SLEDAI* systemic lupus erythematosus disease activity index, *TLR* toll-like receptor, *Treg* regulatory T lymphocytes, *ZBMI* *Z* score body mass index

#### Juvenile idiopathic arthritis

Juvenile idiopathic arthritis (JIA) is a heterogeneous group of chronic arthritides with clinical manifestation starting before the age of 16 years, comprising seven subtypes. The symptoms are predominantly related to inflammation of peripheral articulations but also can involve axial joints, as well as extra-articular structures such as the uvea, skin, entheses, bursae, and internal organs. The prevalence of JIA varies between 16 and 150 per 100,000 people, making the disease the most common JRD [61]. The pathogenesis of JIA remains under investigation, but it seems to be related to genetic susceptibility and environmental factors that destabilize immune harmony. JIA subtypes are associated with human leukocyte antigen (HLA) genes, similar to rheumatoid arthritis, but also with non-HLA-related genes [62]. Also, many immunological processes involved in JIA development promote aberrant activation of immune cells and increase production of proinflammatory mediators, mainly tumor necrosis factor alpha (TNF-α), IL-1, IL-6, IL-17, and CXCL9, enhancing synovitis and further bony erosions [63]. Despite the heterogeneity, different subsets of JIA patients are treated similarly. The first-line therapy includes nonsteroidal anti-inflammatory drugs, followed by DMARDs, with methotrexate (MTX) as the drug of first choice in almost all patients. In addition, the growing role of biological DMARDs should be highlighted. Treatment needs to begin as soon as possible to achieve remission or minimize disease activity [64].

Despite the effectiveness of the current treatment, some JIA patients do not achieve sustained remission, prompting the search for new therapeutic options. Also, treatment with MTX can further decrease 25(OH)D concentration, requiring adequate vitamin D supplementation [65]. Vitamin D deficiency has often been reported in children with JIA and may be associated with disease frequency and activity, although conflicting findings exist [62, 66, 67]. A study on sun exposure and JIA risk showed that higher UV radiation doses before diagnosis and sun exposure during pregnancy were associated with lower risk and frequency of JIA, potentially linked to dose-dependent vitamin D synthesis in the skin [68].

Instead, a study by Thorsen et al. reported no association between serum 25(OH)D concentration at birth and the risk of JIA later in life [69]. Similarly, a recent Mendelian randomization study by Clarke and colleagues showed no causal relationship between serum 25(OH)D and JIA [70]. Even so, considering the immunomodulatory properties and potential immune-restoring effects of vitamin D discussed earlier, we aim to explore how it affects JIA treatment and outcomes.

In a placebo-controlled RCT, Tang et al. found that supplementation with 2000 IU of cholecalciferol per day increased serum 25(OH)D concentration but did not significantly affect BMD or disease activity in JIA patients [71]. Observational studies by Nandi et al. and Sengler et al. reported negative correlations between 25(OH)D concentration and disease activity in JIA patients, as well as an increased risk of JIA-related uveitis [72, 73]. However, Çomak et al. found no difference in disease activity or 25(OH)D concentration between JIA patients with and without standard-dose supplementation [74]. Marini and colleagues observed suboptimal 25(OH)D concentration in a majority of JIA patients, with no significant improvement in 25(OH)D concentration in those receiving supplementation. The researchers also identified associations among VDR polymorphisms and JIA susceptibility and serum 25(OH)D concentrations [75].

In a cross-sectional case–control study, Dağdeviren-Çakır et al. found significantly lower serum 25(OH)D concentration in JIA patients than in healthy controls, although no association with disease activity was observed [76]. Similarly, Bouaddi et al. reported a negative correlation between 25(OH)D and DAS-28 for certain JIA subtypes, but without statistical significance [77].

JIA patients often have vitamin D insufficiency or deficiency, and more well-designed RCTs are needed to confirm the potential associations. Rapid supplementation with regular monitoring and dosage adjustments, along with efforts to determine optimal supplementation doses and duration for children with JIA, is recommended.

#### Juvenile systemic lupus erythematosus

The prevalence of systemic lupus erythematosus (SLE) varies geographically, ranging from 29 to 210 per 100,000 people in Europe and from 48 to 366 per 100,000 people in North America [78]. Juvenile SLE (jSLE) is diagnosed in patients younger than 16 years, accounting for approximately 10%–20% of all SLE cases [79]. jSLE and adult SLE (aSLE) have similar symptomatology, pathophysiology, and treatment patterns, but jSLE often presents with a more acute and aggressive clinical course, with higher rates of systemic involvement, particularly in the kidneys, blood, and nervous system [80]. The disease course varies within the pediatric group, with rare cases in patients younger than 5 years, limited symptoms in prepubertal onset, and a peak incidence at age 12–14 years [81].

In jSLE, serological testing shows a higher occurrence of anti-double-stranded DNA antibodies and antibodies against cellular components than in aSLE. However, a larger percentage of jSLE patients do not exhibit antinuclear antibodies, particularly during the prepubertal period [79]. Pathophysiology of jSLE is characterized by increased activation of B and effector T lymphocytes, reduced regulatory T cells, elevated proinflammatory cytokines, and decreased immune-regulating cytokines. Although some mutations increase susceptibility to SLE, most cases of jSLE are not solely attributed to genetic factors, but pediatric patients may have a higher prevalence of gene variants associated with SLE risk [81].

Treating jSLE requires more immune-modulating medications, including glucocorticosteroids (GCS), than for aSLE, because jSLE exhibits higher disease activity measured by the SLE disease activity index [82]. Some studies suggest an inverse correlation between proinflammatory cytokines (IFN-γ and IL-17) and serum 25(OH)D concentration in jSLE patients [83]. Analysis of the literature indicates that jSLE patients are commonly vitamin D deficient [84, 85], and its potential role in treatment has been investigated. A cross-sectional study by Tabra et al. reported lower serum 25(OH)D concentration in jSLE patients, with significant correlations between 25(OH)D concentration and disease activity, steroid dose, and biomarkers [85]. Similar observations were made by Stagi et al., showing correlations between 25(OH)D concentration and various parameters, including BMD [84]. Caetano et al. studied female adolescents with jSLE and found a significant decrease in BMD over time, particularly in patients without vitamin D supplementation [86]. In contrast, Peracchi et al. did not find significant correlations between 25(OH)D concentration and disease activity, PTH, or BMD in jSLE patients, despite a lower concentration of 25(OH)D than in controls [87].

Two RCTs investigated vitamin D’s influence on jSLE. In a double-blind, placebo-controlled trial, Lima et al. administered oral cholecalciferol supplementation (50,000 IU per week) to one group of 40 jSLE patients for 24 weeks [88]. The intervention group showed an increased 25(OH)D concentration and significant improvements in disease activity and fatigue in comparison with the controls. The second RCT from Lima et al. revealed significant improvement in bone microarchitecture, reflected in the increased trabecular number and decreased trabecular separation, after a weekly dose of 50,000 IU of cholecalciferol administered for 24 consecutive weeks compared to placebo [89].

Vitamin D deficiency is prevalent in jSLE patients and may contribute to the onset of the disease. Despite jSLE’s more aggressive nature and the need for anti-inflammatory medications, including GCS, vitamin D supplementation is a reasonable addition to the treatment regimen. However, the reviewed studies used fixed doses of vitamin D and did not achieve adequate intake in all patients, complicating efforts to determine the optimal dosage for jSLE treatment. Certainly, further well-designed trials are needed to explore vitamin D’s immunomodulatory role, because current data are limited.

#### Juvenile systemic scleroderma

Juvenile systemic scleroderma (jSSc) is an inflammatory connective tissue disorder with various degrees of multisystemic involvement diagnosed in children younger than 16 years. According to the most recent data, the prevalence of jSSc is estimated at 3 cases per million children [90], and the incidence is approximately 0.27 cases per million children. Moreover, less than 10% of all systemic scleroderma cases begin in childhood or adolescence [91]. The pathogenesis is multifactorial and not fully understood. However, both adult-onset and jSSc are believed to share the same underlying mechanisms. The disease arises from dysregulation of both the innate and adaptive immune systems, leading to an imbalance favoring proinflammatory cells and resulting in the overproduction of proinflammatory and profibrotic cytokines, including IL-6, IL-4, IL-1, transforming growth factor beta, and autoantibodies. A significant reduction in T regulatory cells also occurs. Those inflammatory processes affect the microvasculature, promoting vasoconstriction and leading to vascular damage and remodeling. Furthermore, fibroblasts transform into secretory subtypes that produce more collagen and profibrotic cytokines [92–94]. Some studies have identified genetic and environmental factors that may increase susceptibility to the disease [95]. The autoantibody patterns differ between types of SSc. Lower prevalence of antinuclear antibody and anti-topoisomerase I antibody (anti-Scl-70) has been found in jSSc, whereas anticentromere and anti-RNA polymerase III may be more prevalent [96].

Clinical manifestations of jSSc involve the skin (especially the face and hands) and peripheral circulatory disorders such as Raynaud’s phenomenon. Musculoskeletal involvement is common, with symptoms such as joint stiffness and muscle weakness but gastrointestinal, pulmonary, and cardiovascular systems also can be affected [97, 98]. Three main subtypes of jSSc are distinguished: diffuse cutaneous, limited cutaneous, and overlapping [99].

Several studies have highlighted notable differences in progression between juvenile- and adult-onset SSc. According to Adrovic et al., adult patients exhibit a significantly higher frequency of interstitial lung disease than their juvenile counterparts [100]. Although no significant differences were observed regarding renal, gastrointestinal, and cardiac involvement, a significantly higher incidence of arterial hypertension was reported among adults. Conversely, jSSc patients presented a significantly higher incidence of arthralgia and arthritis, but no disparities were observed in other musculoskeletal symptoms. Also, the predominant disease subtype varied significantly, with juvenile patients more commonly diagnosed with the diffuse cutaneous subset of SSc, whereas the limited cutaneous SSc subset was more prevalent among adult patients. Foeldevari and colleagues [101] reported similar findings. Research has shown that adults with diffuse cutaneous SSc have lower levels of serum 25(OH)D than patients with limited cutaneous SSc [102]. Moreover, vitamin D-deficient diffuse SSc patients experience reduced quality of life [103]. Those findings collectively highlight the importance of focusing on jSSc patients and their vitamin D levels.

Distinct treatment patterns were observed between jSSc and adult-onset SSc. MTX and GCS were more often used in jSSc than in adult-onset SSc, whereas no significant differences were noted regarding the use of other DMARDs. Conversely, adult patients more often required calcium channel blockers and angiotensin-converting enzyme inhibitors, potentially due to the higher incidence of organ involvement, including those affecting the cardiovascular system [100, 101, 104].

Although available data confirm that jSSc patients are at risk of vitamin D deficiency [105], no trials investigating the role of vitamin D supplementation in jSSc could be found. The identified studies discussed only cholecalciferol’s theoretical influence on disease progression. Importantly, there are no significant contraindications to administering vitamin D in jSSc, although well-designed RCTs are necessary to determine vitamin D’s potential efficacy in the treatment regimen. Considering the prevalence of vitamin D deficiency in both the general population and, particularly, among patients with these chronic diseases, consistent vitamin D supplementation appears to be a reasonable measure for all patients with jSSc.

#### Juvenile idiopathic inflammatory myopathies

Juvenile idiopathic inflammatory myopathies (JIIMs) are rare autoimmune disorders affecting children and adolescents. JIIMs result from a complex interplay of genetic predisposition, environmental triggers, and immune dysregulation. Persistent muscle inflammation occurs as a result of autoimmune reactions, involving T-cell activation and release of proinflammatory molecules [106, 107]. Genetic factors, including variations in HLA genes, and environmental triggers contribute to susceptibility [106–108]. Ongoing inflammation leads to muscle damage, causing weakness and functional impairment. The exact mechanisms of muscle damage are still under investigation but probably involve immune-mediated destruction and impaired muscle regeneration [109].

Juvenile dermatomyositis (JDM) is the primary form of JIIM, making up about 80% of cases. The annual incidence of JDM in children younger than 16 years ranges from 2.28 to 3.17 per million [106, 110]. Pathogenesis of JDM involves upregulated type I interferon-dependent genes [111]. Anti-p155/140 and anti-MJ antibodies are commonly observed in JDM [106]. JDM patients should be wary of excessive sun exposure because of the aggravating effects of UV radiation. That effect can result in decreased levels of serum 25(OH)D, which correlates with disease activity score as reported by Robinson et al. [112]. Therefore, we advise considering appropriate doses of vitamin D to prevent deficiency in JDM patients.

Overlap myositis, the second-most-common phenotype in JIIM, involves patients with an additional autoimmune disease. The phenotype is observed in approximately 6%–11% of JIIM patients [113]. Those overlapping diseases have been associated with lower 25(OH)D concentration.

Juvenile polymyositis (JPM) occurs in about 4–8% of JIIM cases. It typically presents during adolescence, causing weakness in both proximal and distal muscles, frequent falling episodes, muscle pain (myalgias), and elevated creatine kinase levels. JPM has a more severe onset than JDM, with about 35% of JPM patients experiencing cardiac involvement. Weight loss and Raynaud’s phenomenon also are commonly observed. Distinctive pathological features, including endomysial infiltrates, are seen in affected muscles [106, 113].

Our literature research, conducted based on previously described criteria, found no trials concerning vitamin D supplementation in the pediatric population with idiopathic inflammatory myopathies. Nonetheless, most patients with idiopathic inflammatory myopathies, both adults and children, exhibit low serum 25(OH)D concentrations, which could play a role in developing adult myositis, just similar to certain other autoimmune disorders [112, 114]. By contrast, an in vitro study by Di Luigi et al. revealed that some VDR agonists exhibited strong efficacy in reducing the secretion of CXCL10 protein induced by IFN-γ/TNF-α, showing their potent inhibitory effects [115]. In addition, they targeted the signaling pathways downstream of TNF-α in those cells. The knowledge about immune and nonimmune pathways related to the development of this complex group of diseases is continually growing. Well-designed in vitro and in vivo research is required to shed light on vitamin D’s potential complementary role in treating JIIMs.

#### Behçet disease

Behçet’s disease (BD) is a systemic vasculitis that affects both the arteries and veins, presenting with a relapsing and remitting nature and having a complex pathogenesis [116, 117]. Typical manifestations include recurrent fever, oral and genital aphthae, alongside other features involving joints, eyes, gastrointestinal, and nervous systems [116, 118]. The clinical presentation varies slightly between adult and pediatric patients [119], with male patients experiencing a more severe course [120]. While not common in the pediatric population, specific diagnostic criteria for pediatric BD have been published, requiring full clinical manifestation below the age of 16 years [117]. Originally occurring along the “Silk Road” area, BD is now observed worldwide, primarily due to migrations, whereas the estimated prevalence in children is 1 per 600,000 [121].

Treatment depends on organ involvement and includes colchicine, immunosuppressives, and biologic drugs [119]. Data regarding the status of vitamin D in BD patients are primarily acquired from adult or mixed populations and remain limited. Some authors confirm lower 25(OH)D concentration in BD patients compared to controls [122], while others report opposite results, noting a significant decrease in serum 25(OH)D during active BD [122, 123]. Potential associations between vitamin D and laboratory and clinical features of BD have been largely investigated. For instance, Omar et al. found that decreased vitamin D levels in BD patients were associated with higher concentrations of oxidative stress markers compared to healthy controls [124]. Additionally, Güngör et al. revealed that BD patients with vitamin D deficiency exhibited higher concentrations of endothelial selectin molecules, playing an important role in the disease’s pathogenesis. Interestingly, these patients showed significant improvement after 3-month-long vitamin D supplementation [125]. This could, at least partly, support another observation by Can et al. who demonstrated that supplementation with a daily dose of 1000 IUvitamin D for 3 months significantly improved carotid intima-media thickness [122]. Another observational study showed that insufficient levels of vitamin D were associated with the promotion of Th1 lymphocytes, and a decrease in Treg cells [126]. Similarly, Tian et al. confirmed that vitamin D significantly inhibited the differentiation of Th17 cells in BD patients [127].

Contrary to all of the aforementioned studies, the latest Mendelian randomization study by Zhong et al. revealed a potential link between lifelong higher 25(OH)D concentrations and an increased risk of BD [128]. These findings warrant further research, to elucidate real associations in the specific disease. Unfortunately, no RCTs regarding the therapeutic role of vitamin D in the pediatric BD population have been published so far. More studies in this field are still required to clarify whether vitamin D can play a supplementary role in the treatment of BD in children.

#### Juvenile periodic fever syndromes

Autoinflammatory diseases (AIDs) encompass a diverse group of conditions triggered by the activation of the innate immune system [129]. Unlike autoimmune disorders, AIDs are not associated with autoantibodies or antigen-specific T cells. Many of these diseases are linked to inborn errors of innate immunity, affecting the function of immune cells. While most AIDs are caused by monogenic mutations inherited in an autosomal dominant or recessive pattern, some remain unexplained by mutations in known periodic syndrome-related genes. The exact underlying causes vary and require further investigation, but they often involve specific endogenous or exogenous stimuli [130].

Clinical presentations of most AIDs include recurring episodes of fever, accompanied by increased inflammatory markers, followed by periods of general well-being, with the onset typically in childhood [130]. Among the common pediatric autoinflammatory diseases are periodic fever, aphthous stomatitis, pharyngitis, and adenitis syndrome (PFAPA), familial Mediterranean fever (FMF), tumor necrosis factor (TNF)-receptor-associated periodic syndrome (TRAPS), hyper-IgD syndrome (HIDS), as well as the cryopyrin-associated periodic syndromes (CAPS), and systemic undifferentiated recurrent fever (SURF) [131].

Not only the diagnostics but also the treatment of these rare and underestimated conditions have posed many challenges for physicians, with a revolutionary role of biological treatment blocking specific cytokines, which are oversecreted in AIDs [132].

#### Periodic fever, aphthous stomatitis, pharyngitis and adenitis syndrome

Evidence is limited although some investigators, e.g. Mahamid et al. or Nalbantoğlu et al. reported a notable association between PFAPA outcome and vitamin D deficiency [133, 134]. Furthermore, Stagi et al. demonstrated lower serum 25(OH)D concentrations in patients with PFAPA compared to healthy controls, particularly during the winter months. Moreover, their study showed that daily supplementation of 400 IU of cholecalciferol led to a reduction in the frequency and duration of febrile episodes [135].

#### Familial Mediterranean fever

Previous studies have reported lower levels of vitamin D in children with FMF [136, 137]. Moreover, an interesting study by Dağdeviren-Çakır confirmed that during attack periods in FMF patients, serum 25(OH)D concentration was significantly lower [76]. An interventional study conducted by Kazem et al. demonstrated that a 6-month dietary supplementation regimen, consisting of flaxseed, curcumin, and 4000 IU of daily vitamin D, significantly improved cognitive functions and the course of FMF in terms of attack frequency, severity, and duration among the patient group. However, the study did not assess the effects of the specific supplements individually [138]. Thus, generalizable conclusions regarding the beneficial role of vitamin D in FMF should be drawn and discussed carefully.

Our review did not determine published studies investigating the status or potential role of vitamin D in HIDS, TRAPS, CAPS syndrome, and SURF. The available data examining the relationship between recurrent fever syndromes and vitamin D are scarce, indeed, due to the low prevalence. These conditions are considered to be rare, and—importantly—have a relatively short history of research. Given the limited data availability, the field remains open for future research, particularly in prospective cohorts, and also regarding dose–response studies.

## Summary and conclusions

Vitamin D exhibits a broad range of beneficial effects on various human cells. Its pleiotropic impact includes regulating the immune system and modulating autoimmune disorders, including pediatric rheumatic conditions, as shown by in vitro and in vivo studies. Several investigations have shown an inverse relationship between vitamin D deficiency and disease severity or outcomes in those conditions, although some reports indicate limited effects. Although JRDs are relatively uncommon, they significantly affect the pediatric population’s health and can have long-term negative consequences on quality of life, even into adulthood.

Despite inconsistent evidence, vitamin D is believed to have a positive influence on people with various diseases, including rheumatic conditions. However, published data are scarce regarding vitamin D’s modifying or complementary role in pediatric rheumatology. That lack may be attributed to methodological issues and the flawed design of previous studies, which often resembled drug trials rather than investigations into nutrient effects. Classical skeletal health outcomes related to serum 25(OH)D concentration may be variable and may often be strongly dependent on the initial level i.e. adequacy or deficiency, however, the threshold required to activate all the extraskeletal effects, particularly those related to autoimmune regulation, remains uncertain. Furthermore, most RCTs used the same vitamin D dosage for all participants, without ensuring its efficacy in achieving sufficient serum 25(OH)D concentrations for each individual. Although the data gathered for our review are promising, the findings are insufficient to confirm vitamin D’s supplementary role in treating JRDs.

Therefore, further well-designed interventional studies, especially RCTs, are needed to establish definitive conclusions and develop specific recommendations. Dose–response studies in the field of pediatric rheumatology may be particularly useful. Considering the high prevalence and the growing incidence of vitamin D deficiency, even in the healthy population, we advise encouraging children and adolescents to follow current recommendations for vitamin D supplementation.

## Supplementary Information

Below is the link to the electronic supplementary material.

Supplementary file1 (DOC 51 KB)

## Data Availability

Not applicable to this article as no datasets were generated during the current study.
