# Supplementary material for: Can vitamin D be an adjuvant therapy for juvenile rheumatic diseases?
Source: Rheumatol Int. 2023 Aug 11;43(11):1993–2009. doi: 10.1007/s00296-023-05411-5 (PMC10495493; doi:10.1007/s00296-023-05411-5)
Supplement: Supplementary file 1 — Supplementary file1 (DOC 51 KB) [file 296_2023_5411_MOESM1_ESM.doc]

Appendix

Search strategies carried out on Medline/PubMed, Scopus, and EMBASE databases

**The number of publications found for each inquiry has been given in brackets ()**

*1. Juvenile idiopathic arthritis*

**MEDLINE/PubMed:**

1. “vitamin D AND juvenile idiopathic arthritis” (108)

2. “vitamin D juvenile idiopathic arthritis” (108)

3. “vitamin D & juvenile idiopathic arthritis” (20)

4. 1 OR 2 OR 3 (108)

32 relevant, further investigated publications

**Embase:**

#1. “vitamin D juvenile idiopathic arthritis” (73)

#2. “vitamin D and juvenile idiopathic arthritis” (51)

#3. “vitamin D & juvenile idiopathic arthritis” (73)

#4. 1 OR 2 OR 3 OR 4 OR 5 OR 6 (111)

23 relevant, further investigated publications

**Scopus:**

vitamin D AND in AND juvenile AND idiopathic AND arthritis (157)

35 relevant, further investigated publications

*2. Juvenile systemic lupus erythematosus*

**MEDLINE/PubMed:**

1. “vitamin D AND juvenile systemic lupus erythematosus” (34)

2. “vitamin juvenile systemic lupus erythematosus” (34)

3. “vitamin D & juvenile systemic lupus erythematosus” (4)

4. 1 OR 2 OR 3 (38)

16 relevant, further investigated publications

**Embase:**

#1. “vitamin D juvenile systemic lupus erythematosus” (23)

#2. “vitamin D and juvenile systemic lupus erythematosus” (20)

#3. “vitamin D & juvenile systemic lupus erythematosus” (23)

#4. 1 OR 2 OR 3 (29)

11 relevant, further investigated publications

**Scopus:**

vitamin D AND in AND juvenile AND systemic AND lupus AND erythematosus 101 results

18 relevant, further investigated publications

3. *Juvenile inflammatory myopathies*

**MEDLINE/PubMed:**

1. vitamin D AND juvenile inflammatory myopathies (12)

2. vitamin D juvenile inflammatory myopathies (12)

3. vitamin D & juvenile inflammatory myopathies (8)

4. 1 OR 2 OR 3 (12)

8 relevant, further investigated publications

**Embase:**

#1. “vitamin D juvenile inflammatory myopathies” (11)

#2. “vitamin D and juvenile inflammatory myopathies” (11)

#3. “vitamin D & juvenile inflammatory myopathies” (10)

#4. 1 OR 2 OR 3 (11)

4 relevant, further investigated publications

**Scopus:**

vitamin D AND juvenile AND inflammatory AND myopathies (18)

7 relevant, further investigated publications

4. *Juvenile systemic scleroderma*

**MEDLINE/PubMed:**

1. “vitamin D AND juvenile systemic scleroderma” (6)

2. “vitamin D juvenile systemic scleroderma” (6)

3. “vitamin D & juvenile systemic scleroderma”

4. 1 OR 2 OR 3 (6)

3 relevant, further investigated publications

**Embase:**

#1. “vitamin D juvenile systemic scleroderma” (17)

#2. “vitamin D and juvenile systemic scleroderma” (17)

#3. “vitamin D & juvenile systemic scleroderma” (15)

#4. 1 OR 2 OR 3 (17)

8 relevant, further investigated publications

**Scopus:**

vitamin D AND juvenile AND systemic AND scleroderma (21)

9 relevant, further investigated publications

5. *Juvenile rheumatic diseases*

**MEDLINE/PubMed:**

1. “vitamin D AND juvenile rheumatic diseases” (91)

2. “vitamin D juvenile rheumatic diseases” (91)

3. “vitamin D & juvenile rheumatic diseases” (10)

4. 1 OR 2 OR 3 (91)

33 relevant, further investigated publications

**Embase:**

#1. “vitamin D juvenile rheumatic diseases” (55)

#2. “vitamin D and juvenile rheumatic diseases” (55)

#3. “vitamin D & juvenile rheumatic diseases” (43)

#4. 1 OR 2 OR 3 (55)

18 relevant, further investigated publications

**Scopus:**

vitamin D AND juvenile AND rheumatic AND diseases (70)

25 relevant, further investigated publications

6. *Behcet disease*

**MEDLINE/PubMed:**

1. “vitamin D AND Behcet disease” (50)

2. “vitamin D Behcet disease”(50)

3. 1 OR 2 (50)

20 relevant, further investigated publications

**Embase:**

#1. “vitamin D Behcet disease” (62)

#2. “vitamin D and Behcet disease” (62)

#3. “vitamin D & Behcet disease” (44)

#4. 1 OR 2 OR 3 (62)

19 relevant, further investigated publications

**Scopus:**

vitamin D AND Behcet AND disease (109)

23 relevant, further investigated publications

7. *Periodic fever aphthous stomatitis pharyngitis and adenopathy syndrome (PFAPA)*

**MEDLINE/PubMed:**

1. “vitamin D AND periodic fever aphthous stomatitis pharyngitis and adenopathy syndrome” (1)

2. “vitamin D periodic fever aphthous stomatitis pharyngitis and adenopathy syndrome” (1)

3. “vitamin D AND PFAPA” (6)

4. “vitamin D PFAPA” (6)

5. 1 OR 2 OR 3 OR 4 (7)

7 relevant, further investigated publications

**Embase:**

#1. “vitamin D periodic fever aphthous stomatitis pharyngitis and adenopathy syndrome” (3)

#2. “vitamin D and periodic fever aphthous stomatitis pharyngitis and adenopathy syndrome” (3)

#3. “vitamin D & periodic fever aphthous stomatitis pharyngitis and adenopathy syndrome” (1)

#4. “vitamin D PFAPA” (7)

#5. “vitamin D and PFAPA” (7)

#6. “vitamin D & PFAPA” (2)

#7. 1 OR 2 OR 3 OR 4 OR 5 OR 6 (7)

5 relevant, further investigated publications

**Scopus:**

1. vitamin D AND periodic AND fever AND aphthous AND stomatitis AND pharyngitis AND adenopathy AND syndrome (1)

2. vitamin D AND PFAPA (13)

8 relevant, further investigated publications

8. *Familial Mediterranean fever (FMF)*

**MEDLINE/PubMed:**

1. “vitamin D AND familial Mediterranean fever” (20)

2. “vitamin D familial Mediterranean fever” (20)

3. “vitamin D AND FMF” (15)

4. “vitamin D FMF” (15)

5. 1 OR 2 OR 3 OR 4 (20)

17 relevant, further investigated publications

**Embase:**

#1. “vitamin D familial Mediterranean fever” (31)

#2. “vitamin D and familial Mediterranean fever” (31)

#3. ”vitamin D & familial Mediterranean fever” (6)

#4. “vitamin D FMF” (23)

#5. “vitamin D and FMF” (23)

#6. “vitamin D & FMF” (4)

#7. 1 OR 2 OR 3 OR 4 OR 5 OR 6 (31)

15 relevant, further investigated publications

**Scopus:**

1. vitamin D AND familial AND Mediterranean AND fever (45)

2. vitamin D AND FMF (28)

19 relevant, further investigated publications

9. *Hyper-IgD syndrome (HIDS)*

**MEDLINE/PubMed:**

1. “vitamin D AND hyper-IgD syndrome” (0)

2. “vitamin D hyper-IgD syndrome” (0)

3. “vitamin D AND HIDS” (0)

4. “vitamin D HIDS” (0)

5. 1 OR 2 OR 3 OR 4 (0)

0 relevant, further investigated publications

**Embase:**

#1. “vitamin D AND hyper-IgD syndrome” (0)

#2. “vitamin D hyper-IgD syndrome” (0)

#3. “vitamin D & hyper-IgD syndrome” (0)

#4. “vitamin D AND HIDS” (2)

#5. “vitamin D HIDS” (2)

#6. “vitamin D & HIDS” (0)

#7. 1 OR 2 OR 3 OR 4 OR 5 OR 6 (2)

0 relevant, further investigated publications

**Scopus:**

1. vitamin D AND hyper-IgD AND syndrome (0)

2. vitamin D AND HIDS (2)

0 relevant, further investigated publications

10. *Cryopyrin-associated periodic syndrome (CAPS)*

**MEDLINE/PubMed:**

1. “vitamin D AND cryopyrin-associated periodic syndrome” (1)

2. “vitamin D cryopyrin-associated periodic syndrome” (1)

3. “vitamin D AND CAPS” (13)

4. “vitamin D CAPS” (13)

5. 1 OR 2 OR 3 OR 4 (14)

0 relevant, further investigated publications

**Embase:**

#1. “vitamin D cryopyrin-associated periodic syndrome” (0)

#2. “vitamin D and cryopyrin-associated periodic syndrome” (0)

#3. “vitamin D & cryopyrin-associated periodic syndrome” (0)

#4. “vitamin D CAPS”(101)

#5. “vitamin D and CAPS” (101)

#6. “vitamin D & CAPS” (11)

#7. 1 OR 2 OR 3 OR 4 OR 5 OR 6 (101)

0 relevant, further investigated publications

**Scopus:**

1. vitamin D AND cryopyrin-associated AND periodic AND syndrome (3)

2. vitamin D AND CAPS (198)

0 relevant, further investigated publications

11. *Tumor necrosis factor receptor-associated periodic syndrome (TRAPS)*

**MEDLINE/PubMed:**

1. “vitamin D AND tumor necrosis factor receptor-associated periodic syndrome” (0)

2. “vitamin D tumor necrosis factor receptor-associated periodic syndrome” (0)

3. “vitamin D AND TRAPS” (54)

4. “vitamin D TRAPS” (54)

5. 1 OR 2 OR 3 OR 4 (55)

0 relevant, further investigated publications

**Embase:**

#1. “vitamin D tumor necrosis factor receptor-associated periodic syndrome” (0)

#2. “vitamin D and tumor necrosis factor receptor-associated periodic syndrome”(0)

#3. “vitamin D & tumor necrosis factor receptor-associated periodic syndrome” (0)

#4. “vitamin D TRAPS” (217)

#5. “vitamin D and TRAPS” (217)

#6. “vitamin D & TRAPS” (101)

7. 1 OR 2 OR 3 OR 4 OR 5 OR 6 (217)

0 relevant, further investigated publications

**Scopus:**

1. vitamin D AND tumor AND necrosis AND factor AND receptor-associated AND periodic AND syndrome (3)

2. vitamin D AND TRAPS (311)

0 relevant, further investigated publications

12. *Systemic undifferentiated recurrent fever (SURF)*

**MEDLINE/PubMed:**

1. “vitamin D AND systemic undifferentiated recurrent fever” (0)

2. “vitamin D systemic undifferentiated recurrent fever” (0)

3. “vitamin D AND SURF” (4)

4. “vitamin D SURF” (4)

5. 1 OR 2 OR 3 OR 4 (4)

0 relevant, further investigated publications

**Embase:**

#1. “vitamin D systemic undifferentiated recurrent fever” (0)

#2. “vitamin D and systemic undifferentiated recurrent fever” (0)

#3. “vitamin D & systemic undifferentiated recurrent fever” (0)

#4. “vitamin D SURF” (217)

#5. “vitamin D and SURF” (217)

#6. “vitamin D & SURF” (101)

#7. 1 OR 2 OR 3 OR 4 OR 5 OR 6 (217)

0 relevant, further investigated publications

**Scopus:**

1. vitamin D AND systemic AND undifferentiated AND recurrent AND fever (0)

2. vitamin D AND SURF (4)

0 relevant, further investigated publications

13. *Periodic fever syndromes*

**MEDLINE/PubMed:**

1. “vitamin D AND periodic fever syndromes” (15)

2. “vitamin D periodic fever syndromes” (15)

3. 1 OR 2 (15)

6 relevant, further investigated publications

**Embase:**

#1. “vitamin D periodic fever syndromes” (12)

#2. “vitamin D and periodic fever syndromes” (12)

#3. “vitamin D & periodic fever syndromes” 4)

#4. 1 OR 2 OR 3 (12)

3 relevant, further investigated publications

**Scopus:**

vitamin AND d AND periodic AND fever AND syndromes (18)

7 relevant, further investigated publications
